# Changes in the serum proteome associated with the development of hepatocellular carcinoma in hepatitis C-related cirrhosis

**DOI:** 10.1038/sj.bjc.6602923

**Published:** 2006-01-10

**Authors:** D G Ward, Y Cheng, G N'kontchou, T T Thar, N Barget, W Wei, L J Billingham, A Martin, M Beaugrand, P J Johnson

**Affiliations:** 1 Cancer Research UK Institute for Cancer Studies, School of Medicine, University of Birmingham, Edgbaston, Birmingham B15 2TT, UK; 2 Hepto-gastroenterology and Pathology Department, Jean Verdier Hospital, Assistance Publique-Hospitaux de Paris, UPRES EA 3409, UFR SMBH, Université Paris 13, Bondy, France

**Keywords:** hepatocellular carcinoma, serum, proteome, SELDI

## Abstract

Early diagnosis of hepatocellular carcinoma (HCC) is the key to the delivery of effective therapies. The conventional serological diagnostic test, estimation of serum alpha-fetoprotein (AFP) lacks both sensitivity and specificity as a screening tool and improved tests are needed to complement ultrasound scanning, the major modality for surveillance of groups at high risk of HCC. We have analysed the serum proteome of 182 patients with hepatitis C-induced liver cirrhosis (77 with HCC) by surface-enhanced laser desorption/ionisation time-of-flight mass spectrometry (SELDI). The patients were split into a training set (84 non-HCC, 60 HCC) and a ‘blind’ test set (21 non-HCC, 17 HCC). Neural networks developed on the training set were able to classify the blind test set with 94% sensitivity (95% CI 73–99%) and 86% specificity (95% CI 65–95%). Two of the SELDI peaks (23/23.5 kDa) were elevated by an average of 50% in the serum of HCC patients (*P*<0.001) and were identified as *κ* and *λ* immunoglobulin light chains. This approach may permit identification of several individual proteins, which, in combination, may offer a novel way to diagnose HCC.

Hepatocellular carcinoma (HCC) is the fifth commonest cancer in the world today and the overall 5-year survival rate remains less than 5% (Parkin *et al*, 2001). Although the incidence rate is likely to fall with the institution of mass vaccination against the hepatitis B virus, initiated in the 1980s (Chang *et al*, 1997), this will not have a major impact for many years as the age of presentation is over 50 years in most areas of the world. Furthermore, there is no prospect of a vaccine against the hepatitis C virus, the major aetiological factor for HCC in the US, Japan and Southern Europe (Di Bisceglie *et al*, 1991; El-Sarag and Hashem, 2004). Despite the absence of randomised clinical trials, there is strong evidence that surgical resection, liver transplantation or ablative therapies significantly improve survival (Bruix *et al*, 2001; Beaugrand *et al*, 2005). Such approaches are, however, only applicable to those in whom the tumour is detected at an early stage, typically less than 3 cm in diameter without vascular involvement and tumours only rarely present with symptoms at this stage (Mazzaferro *et al*, 1996; Bruix *et al*, 2001; Beaugrand *et al*, 2005). Early diagnosis has, therefore, become a priority. Surveillance of high-risk groups, such as those with cirrhosis, has been shown to permit detection of small tumours and there is emerging evidence that this is associated with improved survival (Sherman, 2005). Currently, surveillance involves both serological testing with serum alpha-fetoprotein (AFP) estimation and ultrasound scanning, typically at 6 monthly intervals (Mazzaferro *et al*, 1996; Trevisani *et al*, 2002). However, while AFP may be a useful diagnostic serum marker in patients with advanced symptomatic disease, it is much less useful in patients with earlier/small tumours where its sensitivity is low (Mazzaferro *et al*, 1996; Johnson, 2001; Trevisani *et al*, 2002; Sherman, 2005).

Ultrasound is far more sensitive (in the order of 80%) but it is highly operator dependent (Trevisani *et al*, 2002; Sherman, 2005). More specific and sensitive serological tests, to complement ultrasound scanning, would be of great clinical value.

Surface-enhanced laser desorption/ionisation time-of-flight mass spectrometry (SELDI) has shown potential for cancer biomarker discovery (Adam *et al*, 2002; Li *et al*, 2002; Chen *et al*, 2004; Zhang *et al*, 2004). A subset of the proteome of a biological sample, such as serum, which binds to a specific solid-phase chromatographic surface (known as a ‘protein-chip array’), is subsequently ionised and detected by time-of-flight mass spectrometry. The peak intensities in the SELDI spectra reflect the abundance of proteins and peptides in the serum. The technique is relatively high throughput, allowing samples to be processed in 96-well format at a rate of up to several hundred serum analyses per day per analyst. Various computer-based pattern recognition approaches can then be applied to discriminate between patient groups, for example, those with and without a particular cancer.

SELDI technology has been applied to identify potential serological diagnostic markers for several cancers including ovarian cancer (Zhang *et al*, 2004), prostate cancer (Adam *et al*, 2002), breast cancer (Li *et al*, 2002) and colon cancer (Chen *et al*, 2004). Published studies have indicated that the SELDI approach may also be used to diagnose HCC (Poon *et al*, 2003; Paradis *et al*, 2005; Schwegler *et al*, 2005), although only Paradis *et al* (2005) used an independent test set and gave details on experimental reproducibility. All three studies were based on small numbers of patients with either undefined or late-stage HCC or chronic liver disease arising from several different aetiologies. In addition, there has been little consensus on the proteomic features that are significantly different in the serum of HCC patients.

The present study was confined to patients with cirrhosis and hepatitis C infection, as hepatitis C infection is the most common cause of cirrhosis in the western world, carrying a high risk of HCC. In addition, we chose to study patients with small HCC (3 cm mean diameter, range 1–11 cm) in order to detect early changes that could be usable in a screening situation. Being cognisant of the controversies that surround the use of SELDI technology to identify biomarkers (Baggerly *et al*, 2004; Diamandis, 2004; Ransohoff, 2004), we were particularly careful to address issues of reproducibility and validity. We used a ‘training’ data set to develop artificial neural networks that permitted classification of patients as HCC or non-HCC and then applied these networks to a ‘blind’ test data set. In addition, we purified and identified two of the most discriminatory proteomic features with *m*/*z* ratios of 23 000 and 23 500.

## MATERIALS AND METHODS

### Sample collection

Serum samples were collected between May 1994 and January 2005 at Jean Verdier Hospital, Bondy, France. Sample collection was officially registered and all patients gave informed consent. Sera were stored at −80°C. All patients tested positive for hepatitis C antibodies and hepatitis C RNA on the day of sampling. Hepatocellular carcinoma was diagnosed histologically or noninvasively, according to the Barcelona criteria (Bruix *et al*, 2001). Samples were transported on dry ice to the University of Birmingham, UK, for analysis in February 2005, defrosted (on ice) and multiple 20 *μ*l aliquots taken and stored at −80°C pending SELDI analysis. Quality control (QC) samples were prepared by mixing equal volumes of serum from 27 healthy individuals and stored as multiple aliquots at −80°C.

### Study design

The study consisted of 84 non-HCC patients and 60 HCC patients (the training set) and 38 samples where the classification of HCC/non-HCC identities was not revealed to the UK-based proteomics team (the blind test set) (Table 1). Independent duplicate SELDI spectra were collected for all serum samples using Cu^2+^-loaded IMAC30 protein-chip arrays. Samples were processed using six 96-well bioprocessors over a 2-week period. Three spots per bioprocessor were devoted to identical QC samples, one spot to a 0–20 kDa calibration mix and one spot to a 20–200 kDa calibration mix. Block randomisation was utilised: to the eight spots on each protein-chip array, we applied in random sequence three serum samples from patients without HCC, three serum samples from patients with HCC and two serum samples from the blind test set or one serum sample from the blind test set and either one QC sample or calibration mix. All samples were analysed once on bioprocessors 1–3 and then a separate aliquot of each patient's serum was analysed a second time on bioprocessors 4–6, ensuring that the measurement duplicates were not processed on the same day.

### Surface-enhanced laser desorption/ionisation procedure

An initial experiment using pooled sera from HCC and non-HCC patients was conducted to decide whether H50, CM10, Q10 or Cu^2+^-loaded IMAC30 protein-chip arrays were best able to detect changes in the serum proteome characteristic of HCC. The Cu^2+^-loaded IMAC30 performed best both in terms of the total number of peaks detected and the number of peak intensities that were significantly different between the HCC and non-HCC pooled samples. We proceeded to analyse all of the patient's samples in duplicate using Cu^2+^-loaded IMAC30 protein-chip arrays. The protein-chip arrays were placed in a 96-well bioprocessor and prepared by a 5 min incubation with 50 *μ*l of 100 mM CuSO_4_ followed by a water rinse and 3 × 10 min equilibrations with 200 *μ*l of binding buffer (100 mM NaCl, 500 mM NaH_2_PO_4_/NaOH (pH 7.0)). Serum samples were defrosted on ice and diluted five-fold with 9 M urea, 2% CHAPS, 50 mM Tris/HCl (pH 9.0). Following a brief vortex, the samples were left on ice for 30 min prior to a 10-fold dilution in binding buffer. These 50-fold final dilution samples were loaded on the bioprocessor (100 *μ*l per spot) and incubated at room temperature for 1 h with shaking at 900 r.p.m. After this period, the nonbound material was discarded and the protein-chip arrays were washed (4 × 10 min incubations with 200 *μ*l of binding buffer followed by a water rinse). The protein-chip arrays were allowed to dry for 30 min prior to addition of 1 *μ*l of 50% saturated sinapinic acid in 50% acetonitrile, 0.5% trifluoroacetic acid. The spots were then allowed to dry for another 30 min prior to a second 1 *μ*l addition of sinapinic acid. The protein-chip arrays were analysed in a PBS IIc protein chip reader equipped with an autoloader (Ciphergen, UK). Spectra were collected over 0–20 and 0–200 kDa ranges (488 laser shots) using laser intensities of 165 and 210, respectively. Spectra were externally calibrated in the 0–20 kDa range using all-in-one peptide standard (Ciphergen) with added cytochrome *c* and myoglobin (Sigma). The 0–200 kDa range was calibrated using chymotrypsinogen, bovine serum albumin and phosphorylase b (Sigma). Spectra were normalised using the total ion current from 2 to 20 and 20 to 200 kDa. Peaks were selected and clustered using Biomarker Wizard software (Ciphergen) with the signal to noise ratio >5 for the first pass and >2 for the second, a cluster mass window of 0.2%, and a requirement for peaks to be present in >20% of the spectra. The peak intensities from the duplicate spectra from each patient were averaged and the resulting peak intensities of the 60 HCC patients and 84 non-HCC patients in the training set were compared by two-sample *t*-test and the area under the receiver operator characteristic (ROC) curve used to assess the discriminatory power of each peak.

### Sample classification

Artificial neural networks (ANNs) were used to build committee models to classify serum samples into HCC and non-HCC groups using different numbers of significant peaks. The feed-forward neural networks consisted of three layers: an input layer, a hidden layer and an output layer. The number of input nodes was determined by the number of significant peaks from which the models were trained. The hidden layer connected the input and output layers, and the number of nodes in this layer controlled the complexity and performance of the neural networks. The output layer consisted of a single node whose output was used to classify sample status, representing HCC or non-HCC. The ANN had full connection from the input nodes to the hidden nodes and from the hidden nodes to the output node. All of the connection weights were randomly initialised in the range (−1, +1). The ANNs were trained using the back propagation algorithm. In the procedure of training a committee model, a 10-fold cross-validation approach was used to reduce the risk of ‘over fit’ (Khan *et al*, 2001). The training data set was randomly partitioned into 10 subvalidation sets (10%) and 10 subtraining sets (90%). Each sample was contained only once in the subvalidation sets. Thus, 10 different ANNs were combined to create a committee model. A stepwise approach was used in which many committee models were built using various numbers of the most significant peaks. Significant peaks were identified by two-sample *t*-test if *P* is less than 0.0123 (determined by a false discovery rate of 10%) (Benjamini and Hochberg, 1995). The classification of the blind test set was made according to the majority decision of the six best committee models.

### Biomarker purification and identification

Two pooled samples were prepared, one containing serum from five HCC patients with high SELDI intensity at 23/23.5 kDa and one containing serum from five non-HCC patients with low SELDI intensity at 23/23.5 kDa. These two samples were diluted four-fold with 9 M urea, 2% CHAPS, 50 mM Tris/HCl (pH 9.0) and applied to Q Ceramic HyperD F anion exchange resin. Proteins were eluted stepwise from the resin using buffers at pH 9, 7, 5, 4, 3 and an organic wash. The proteins in these fractions were monitored by SELDI using Cu^2+^-loaded IMAC30 protein-chip arrays and the fractions containing the 23/23.5 kDa biomarkers were applied to a monolithic C18 RP-HPLC column (BeckmanCoulter PF-2D system) and eluted with an acetonitrile gradient in 0.1% trifluoroacetic acid at a flow rate of 0.75 ml min^−1^. Fractions were collected (0.6 min) and analysed by SELDI on Cu^2+^-loaded IMAC30 protein-chip arrays. Fractions containing the 23/23.5 kDa peak were concentrated and further purified by non-reducing 12% SDS–PAGE using MES running buffer (Invitrogen). The bands corresponding to the 23/23.5 kDa biomarkers were excised and washed in 40 mM ammonium bicarbonate/50% acetonitrile. The gel slices were then treated with 50 mM DTT in 40 mM ammonium bicarbonate/10% acetonitrile (1 h at 60°C) followed by 100 mM iodoacetamide (30 min at room temperature in the dark). After several washes with 40 mM ammonium bicarbonate/10% acetonitrile, 20 *μ*l of 12.5 *μ*g ml^−1^ sequencing grade trypsin (Promega) was added to the dried gel bands and digestion allowed to proceed at 37°C overnight. Peptides were extracted with 100 *μ*l of 3% formic acid and analysed by LC-MS/MS using a ThermoFinnigan LCQ Deca XP Plus Ion-Trap linked directly to an LC Packings/Dionex Ultimate nanobore HPLC system. MS/MS data were searched against a database of nonredundant human protein sequences extracted from NCBI using SEQUEST. Data were filtered using Xcorr values of 1.5, 2 and 2.5 for singly, doubly and triply charged parent ions, respectively, and only first hits were considered.

## RESULTS

### Surface-enhanced laser desorption/ionisation quality control

**Figure 1:**
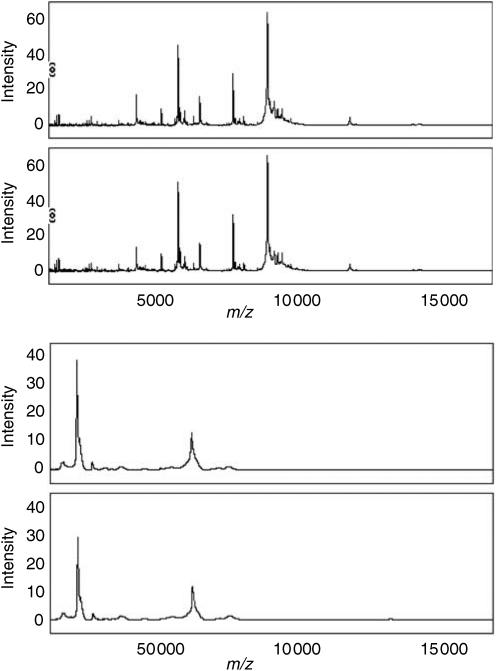
Duplicate low (0–20 000 *m*/*z*) and high (20 000–200 000 *m*/*z*) spectra from a single HCC patient. The spectra were collected 13 days apart on different bioprocessors as described in the Materials and Methods section.

Quality control samples were run in triplicate on all six bioprocessors. ANOVA analysis in Biomarker Wizard software provided no evidence of significant differences between the QC data from different bioprocessors. The coefficient of variation (CV) of the 18 intensities measured for each proteomic feature was calculated and averaged across the 35 most intense peaks. This yielded an average CV of 20±8% (mean±s.d.) obtained during the course of the survey, consistent with the manufacturer's specification (15–20%). Visual inspection of the data revealed no gross differences between duplicate spectra from each patient's serum samples and no data had to be discarded on this basis. An example of duplicate 0–20 and 0–200 kDa spectra for one HCC patient is shown in Figure 1.

A number of patient's samples were haemolysed giving rise to atypical spectra. These were characterised by high haemoglobin peaks (15.1 and 15.9 kDa) and/or a low albumin peak (66.5 kDa). We discarded 34 samples from further data analysis on the basis of a SELDI intensity >5 at 15.9 kDa or <5 at 66.5 kDa. The haemolysed samples were distributed evenly among the HCC and non-HCC patients.

### Significant differences between the serum of HCC and non-HCC patients

Of the 138 peaks picked and clustered by the Biomarker Wizard software, 17 were significantly different at *P*<0.0123 (corresponding to a false discovery rate of 10%) and these are shown in Table2. These peaks have areas under the ROC curve ranging from 0.58 to 0.71 indicating possible diagnostic utility, especially if several of these peaks could be used to build a classifier.

### Artificial neural networks

A total of 17 ANN committee models were developed using up to 17 of the most significant peaks in the training set (*P*<0.0123). The best performing committee models were selected by their ability to correctly assign samples as HCC or non-HCC by 10-fold cross-validation of the training set. A total of 170 ANNs were trained (10 for each committee model). The committee models using the most significant 4, 7, 10, 11, 15 and 17 features were selected with average misclassification rates of 10–15% (cross-validation). Rather than using individual committee models, the majority vote from these six committee models was used to classify the blind test set. It should be emphasised that the blind test set (the key to which was held exclusively by the Bondy group) was only unblinded when the classification model had been finalised, hence operator bias can be excluded from the success of the ANN classification.

**Figure 2:**
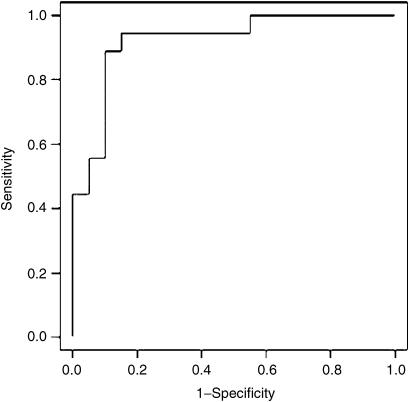
Receiver operator characteristic curve for the ANN classificication of samples in the blind test set.

The blind test set consisted of 17 HCC and 21 non-HCC patients. The majority vote of our ANN committee models correctly predicted 16 HCC patients and 18 non-HCC patients giving 94% sensitivity (95% confidence interval 73–99%, calculated according to Wilson, 1927) and 86% specificity (95% confidence interval 65–95%). Interestingly, all 10 HCC patients in the blind test set that had tumours less than 30 mm were correctly identified, indicating that SELDI can detect liver tumours at an early stage. One of the non-HCC patients diagnosed as having HCC by our analytical approach developed a 25 mm diameter tumour within 6 months of the sample being taken. The area under the ROC curve for the ANN prediction of the blind test set was 0.92 (Figure 2) comparing favourably with 0.73 for AFP (calculated across the whole study).

### Biomarker identification

**Figure 3:**
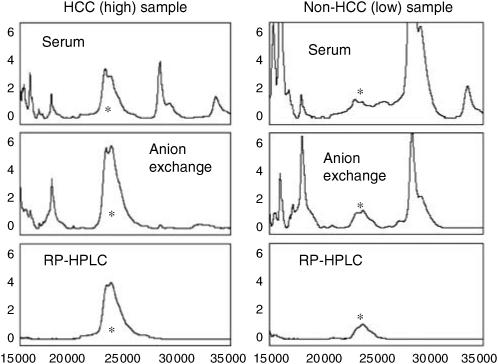
SELDI spectra following the 23/23.5 kDa biomarker purification. Pooled HCC and non-HCC sera, ‘high’ and ‘low’ in the biomarker, respectively, were purified in parallel. Spectra for the original pooled sera, the pH 9 fraction from the anion exchange separation and the peak fraction from the RP-HPLC separation (fraction 25, 0.6 min) are shown. ^*^Indicates the 23/23.5 kDa peak. All spectra were collected using Cu ^2+^-loaded IMAC chips.

**Figure 4:**
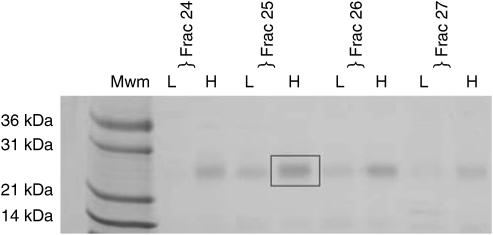
Coomassie stained 12% SDS–PAGE of fractions 24–27 (each 0.6 min) of the RP-HPLC of pooled HCC sera high (H) and pooled non-HCC sera low (L) in the 23/23.5 kDa biomarker. Mwm=molecular weight markers. The rectangle encloses the band that provided the tryptic peptides listed in Table 3.

We selected a broad proteomic feature with peaks at *m*/*z* ratios of 22 960 and 23 530 (hereafter referred to as the ‘23/23.5 kDa peak’) that was significantly elevated in the serum of HCC patients compared to those with cirrhosis alone as a suitable candidate for purification and identification. Of the 138 SELDI peaks, these two were the best discriminators for HCC in this cohort of patients with the exception of a peak with an *m*/*z* ratio of 132 200. Although not formally identified, this peak copurifies with albumin and most likely represents a dimer of albumin. The 23/23.5 kDa peak was purified in parallel from a pool of HCC sera with high intensity at 23/23.5 kDa and a pool of non-HCC sera with low intensity at 23/23.5 kDa (Figure 3). These sera were denatured with urea at pH 9 and applied to anion exchange resin. A double peak at 23/23.5 kDa was found in the resin flow-through (‘pH 9 fraction’) from the ‘high’ sample but was less intense in the pH 9 fraction from the ‘low’ sample indicating that our biomarker is a basic protein that does not bind to the resin under these conditions. The two pH 9 fractions, enriched in the 23/23.5 kDa peak, were applied to an RP-HPLC column and proteins eluted with an acetonitrile gradient. The 23/23.5 kDa peak was detected by SELDI in fractions corresponding to 60–70% acetonitrile and again was more intense in the ‘high’ sample (Figure 3). The fractions containing the 23/23.5 kDa peak were concentrated by centrifugal evaporation and the proteins separated by SDS–PAGE (Figure 4). A band with ∼23 kDa mobility that was more intensely stained in the ‘high’ sample (Figure 4) was excised and trypsinised.

**Figure 5:**
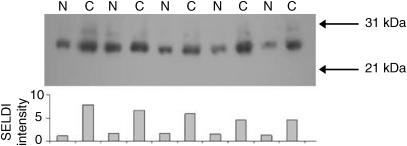
Anti-human IgG blot. Proteins were separated by SDS–PAGE on a 4–12% slab gel and blotted on to PVDF membrane. The blot was probed using horseradish peroxidase-conjugated rabbit polyclonal anti-human IgG antibody (Abcam). The blot shows alternating lanes of non-HCC (N) and HCC (C) sera. The histogram shows the SELDI intensity of each sample at *m*/*z* 22960.

LC-MS/MS identified 34 tryptic peptides of immunoglobulin *κ* light chain and eight peptides from immunoglobulin *λ* light chain. Several homologous peptides, each with a unique mass but reflecting the same part of the sequence of different *κ* or *λ* light chains were seen. For example, 12 homologous peptides were found corresponding to the N-terminal tryptic fragment of *κ* light chain. The data are summarised in Table 3. For each of the seven sets of homologous peptides for the *κ* light chain and five for the *λ* light chain, we have provided the sequence with the highest Xcorr. The peptides listed in Table 3 cover 55 and 34% of the sequence of *κ* and *λ* light chains, respectively, assuming a polypeptide length of 215 amino acids. The multiplicity of peptides demonstrates that the 23/23.5 kDa peak represents a diverse repertoire of immunoglobulin light chains, consistent with both the broad elution from the RP-HPLC column and the broad peak(s) in the SELDI spectra. The identity of the 23/23.5 kDa biomarker was confirmed using an anti-human IgG polyclonal antibody to probe a blot of a SDS–PAGE gel of samples with high and low intensities of the biomarker (Figure 5). The anti-IgG antibody detects more immunoglobulin protein with an electrophoretic mobility of 20–30 kDa (light chains) in the serum samples with greater SELDI intensity at 23/23.5 kDa (Figure 4). The anti-IgG antibody also showed increased binding to proteins of 50 and 100–200 kDa in the samples with greater SELDI intensity at 23/23.5 kDa (data not shown). This suggests that there is an overall increase in the level of IgG in the serum of HCC patients. It is possible that the SELDI peaks at 54 and 149 kDa, upregulated in HCC patients (Table 2), represent immunoglobulin heavy chains and intact IgG, respectively.

## DISCUSSION

In this study, we have shown that SELDI spectra are reproducible and capable of detecting differences in the serum proteome associated with HCC. Artificial neural networks developed using a training set of 84 non-HCC and 60 HCC patients were able to classify a blind test set of 21 non-HCC and 17 HCC patients with 94% sensitivity and 86% specificity. The accuracy of our ANN-based classification is far higher than the currently accepted biomarker, AFP (Gupta *et al*, 2003) (on the patient cohort used in this study, AFP>20 ng ml^−1^ gave 46% sensitivity and 89% specificity). However, this is only a phase 1 biomarker discovery study (Pepe *et al*, 2001). Validation requires analysis of larger cohorts of patients collected at multiple sites. Some early SELDI-based biomarker studies have been heavily criticised for potential bias in their design. This may arise from collection of the cancer and noncancer sera in a different manner, differing storage conditions or poor experimental design, for example, temporal separation between the analysis of the cancer and noncancer samples. In addition, the small sample numbers and high-dimensionality of the data require that care is taken not to overfit data. We have addressed these issues in our experimental design: QC samples were run on all bioprocessors to exclude trends in the protein-chip array reader's performance, block randomisation ensured that any variations in chip quality did not bias the study between HCC and non-HCC or between training and validation sets and the proteomic team in the UK that analysed the samples were unaware of the identities of the blind test set: the ‘key’ was held in France until the patient classification had been completed. In addition, the patient groups were well matched with regard to age (Table 1) and the male/female ratio was well balanced in the test set. Although the male/female ratio was not as well balanced in the training set, only two of the 17 peaks used to build ANNs were significantly different between male and female patients (*m*/*z* 4795 and 66 480, *P*<0.05). Therefore, we can be confident that the proteomic changes we have identified are related to HCC. They could be either proteins secreted by the tumour or arising from secretion from the tumors, induced by inflammatory or immunological response to the tumour or the hallmark of predisposing factors to HCC occurrence. The 23/23.5 kDa peak that we have identified as immunoglobulin light chains may fall into the latter category: IgG levels are higher in patients with more advanced chronic liver disease, itself a predictive factor for HCC.

Previous reports have provided evidence that HCC produces changes in the serum proteome of chronic liver disease patients that can be detected by SELDI (Poon *et al*, 2003; Paradis *et al*, 2005; Schwegler *et al*, 2005). Schwegler *et al* (2005) used SELDI to analyse the serum of 50 hepatitis C patients (28 with HCC). They achieved 61% sensitivity and 76% specificity (using decision trees) and found peaks at 5.8 and 11.7 kDa elevated in HCC. We also see a peak at 5.8 kDa that is upregulated in HCC. Paradis *et al* (2005) were able to classify a set of 82 cirrhotic patients (38 with HCC) with 90% accuracy using logistic regression of data from Zn^2+^-loaded IMAC protein-chip arrays and published a list of 30 proteomic features significantly different between HCC and non-HCC patients. Paradis *et al* did not observe our 23/23.5 kDa biomarker, but some discriminatory features are common to both studies: the intensity of peaks at 33.2 and 66.4 and 102 kDa are decreased (66.4 and 33.2 kDa presumably representing singly and doubly charged ions of albumin). The use of Zn^2+^ rather than Cu^2+^ as the chromatographic ligand and differences in sample preparation and Protein-chip reader settings may account for some differences between the studies in addition to different underlying causes of chronic liver disease and stage of HCC progression. The discriminatory peaks in this study also differ from our earlier work in which serum samples were fractionated prior to SELDI (Poon *et al*, 2003) and again this may be due to the greater mean age and earlier stage of HCC progression in the patients used in our current work. Additionally, the fractionation may have unmasked better discriminators than those observed with whole serum.

It is possible that the improved accuracy of immunoassays over SELDI ‘quantitation’ could improve the performance of biomarkers originally identified by SELDI. The use of multiple carefully chosen markers should enhance both the sensitivity and specificity of HCC screening. Interestingly, unpublished pilot SELDI studies in our laboratory using Cu^2+^-loaded IMAC30 protein-chip arrays also indicate a 23/23.5 kDa peak that is elevated by ∼20% in the serum of colorectal cancer patients (with respect to healthy controls), but not in the serum of oral, breast or prostate cancer patients.

## Figures and Tables

**Table 1:** Patient demographics

**Patient group**	**No. patients**	**Male/female**	**Mean age (s.d.)**
Non-HCC (training set)	84	44/40	65.4 (11.7)
HCC (training set)	60	41/19	71.8 (15.3)
Non-HCC (test set)	21	8/13	67.4 (12.2)
HCC (test set)	17	7/10	68.1 (10.0)

The table shows the number, sex and mean age of the patients analysed in this study. HCC=hepatocellular carcinoma.

**Table 2:** Proteomic features most significantly different between HCC and non-HCC patients

**Peak (*m*/*z*)**	***P* (*t*-test)**	**Area under ROC curve**	**Ratio of mean intensity of HCC sera relative to non-HCC sera**
132 200	1 × 10^−5^	0.709	0.64
23 530	5 × 10^−5^	0.708	1.50
22 960	0.0008	0.693	1.46
53 830	0.0024	0.619	1.43
5254	0.0053	0.650	1.37
33 350	0.0055	0.634	0.90
2362	0.0060	0.619	0.69
3088	0.0064	0.614	0.59
5811	0.0064	0.608	1.67
10 270	0.0072	0.584	0.72
149 410	0.0081	0.625	1.30
66 480	0.0084	0.630	0.90
2273	0.0085	0.634	0.70
94 710	0.0088	0.641	0.85
2792	0.0089	0.606	0.68
4795	0.0119	0.608	1.26
5826	0.0121	0.599	1.51

The table shows the 17 most significant proteomic features based on a two-sample *t*-test. For each feature, we show the mass/charge (*m*/*z*) ratio, *P*-value, area under the receiver operator characteristic (ROC) curve and the mean intensity in the HCC patients relative to the non-HCC patients.

**Table 3:** Peptides identified by LC-MS/MS from the tryptic digest of the 23/23.5 kDa biomarker

**Peptide sequence**	**Xcorr**	**Number of homologous peptides**	**Protein identification**
EIVLTQSPATSLSPGER	4.37	12	Ig*κ* variable region
LLIYGASNLQTGVPSR	2.95	9	Ig*κ* variable region
FSGSNSGNTATLTISR	3.94	5	Ig*κ* variable region
TVAAPSVFIFPPSDEQLK	4.01	3	Ig*κ* constant region
SGTASVVCLLNNFYPR	4.79	1	Ig*κ* constant region
DSTYSLSSTLTLSK	3.67	1	Ig*κ* constant region
VYACEVTHQGLSSPVTKSFNRG	4.65	2	Ig*κ* constant region
YVLTQPPSVSVAPGQTAR	2.62	2	Ig*λ* variable region
SGTSASLAISGLR	3.91	1	Ig*λ* variable region
LTVLSQPK	2.52	3	Ig*λ* variable region
YAASSYLSLTPEQWK	2.95	1	Ig*λ* constant region
SYSCQVTHEGSTVEK	3.31	1	Ig*λ* constant region
